# Redox and Ionic Homeostasis Regulations against Oxidative, Salinity and Drought Stress in Wheat (A Systems Biology Approach)

**DOI:** 10.3389/fgene.2017.00141

**Published:** 2017-10-17

**Authors:** Zahid Hussain Shah, Hafiz M. Rehman, Tasneem Akhtar, Ihsanullah Daur, Muhammad A. Nawaz, Muhammad Q. Ahmad, Iqrar A. Rana, Rana M. Atif, Seung H. Yang, Gyuhwa Chung

**Affiliations:** ^1^Department of Arid Land Agriculture, King Abdulaziz University, Jeddah, Saudi Arabia; ^2^Department of Electronics and Biomedical Engineering, Chonnam National University, Yeosu, South Korea; ^3^Department of Plant Breeding and Genetics, Bahauddin Zakariya University, Multan, Pakistan; ^4^Centre of Agricultural Biochemistry and Biotechnology (CABB), University of Agriculture Faisalabad, Faisalabad, Pakistan; ^5^Department of Plant Breeding and Genetics, University of Agriculture Faisalabad, Faisalabad, Pakistan

**Keywords:** wheat, salinity, drought, oxidative, redox

## Abstract

Systems biology and omics has provided a comprehensive understanding about the dynamics of the genome, metabolome, transcriptome, and proteome under stress. In wheat, abiotic stresses trigger specific networks of pathways involved in redox and ionic homeostasis as well as osmotic balance. These networks are considerably more complicated than those in model plants, and therefore, counter models are proposed by unifying the approaches of omics and stress systems biology. Furthermore, crosstalk among these pathways is monitored by the regulation and streaming of transcripts and genes. In this review, we discuss systems biology and omics as a promising tool to study responses to oxidative, salinity, and drought stress in wheat.

## Introduction

Bread wheat (*Triticum aestivum* L.) is cultivated globally on 200 million ha, with a production of 650 million tons per annum, ranking third after maize and rice (Akpinar et al., 2015). Bread wheat fulfills 20% of the human dietary energy requirement, and is therefore considered a vital component of human diet (Kurtoglu et al., 2014). About 82–85% of the global population depends on wheat for basic food ingredients, i.e., protein, dietary fiber, vitamins, phytochemicals, sugar, and free amino acids (Akpinar et al., 2015). Steady but sustainable increase in wheat yield is an obligatory requirement for future food security (Mochida and Shinozaki, 2013). Every year, environmental stresses such as drought and salinity cause a substantial loss in crop productivity (Wang et al., 2003). Abiotic stresses are a major hindrance to worldwide crop production, and are projected to affect roughly 20% of the total irrigated area worldwide, causing waste to 50% of the land by the mid-21st century (Mahajan and Tuteja, 2005). Of the total available cultivated area of 1.5 billion ha, 20% is irrigated, whereas 60% is rain-fed, contributing to 40 and 60%, respectively, of total food production (Kosová et al., 2014). Wheat, an important crop worldwide, is severely affected by drought stress under both rain-fed and irrigated conditions.

Climatic and various environmental constraints severely influence the production of wheat crop (Semenov et al., 2014). Limited water supply, thermal alterations and high salinity significantly impair the grain yields in wheat (Porter and Semenov, 2005). Potential yield losses associated with these stresses could be combated through the selection and adaptation of cultivars with improved genetic traits (Lobell and Gourdji, 2012; Reynolds et al., 2012). Nevertheless, abiotic stresses not only affect the yield, but also the quality of a crop product, i.e., nutritional value, aroma, color, flavor, and processing properties (Reynolds et al., 2012). Understanding the biochemical pathways, physiological impacts, and molecular mechanisms is highly important to combat these abiotic stresses through the genetic improvements in plants against stress tolerance, yield, and food quality (Hrmova and Lopato, 2014).

Stress biology is a multidisciplinary, integrated, and systematic study of biological systems that utilizes modern omics approaches to analyze genome, metabolome, transcriptome, and proteome under stress (Akpinar et al., 2015). The whole interactome for drought and salinity stress is obtained by the integration of data from relative gene expression, pools of metabolites, and the subsequent production of proteins under stress (Kosová et al., 2014; Kurtoglu et al., 2014). Plants have evolved intricate mechanisms by allowing optimal responses to enable adaptation or avoidance of the stress under such conditions (Hrmova and Lopato, 2014). These intricate mechanisms are usually regulated at the cellular level, such as changes in cell cycle regulation and cell division, membrane adjustments, cell wall modifications, synthesis of endogenic and low-molecular-weight molecules such as abscisic acid, ethylene, jasmonic acid, and salicylic acid (Hrmova and Lopato, 2014; Noctor et al., 2014). All elements that trigger specific mechanisms in response to abiotic stress signals are studied under the scope of stress systems biology (**Table 1**).

**Table 1:** Specific dynamics of stress systems biology associated with abiotic stress signaling.

Molecular processes	Sensing activities	Signaling factors and Receptors	Accessory proteins	Reference
Signaling pathways	Fluctuation in turgor of stomatal guard cells as well as alteration in the levels of cellular K^+^, ABA and pH	PYLs, RCARs, PYR1 (regulatory components of ABA receptors)	MAPK, CIPK	Pizzio et al., 2013; Kosová et al., 2014
Genetic expression and regulation	Rise in the concentration of enzymes responsible for JA biosynthesis Enhanced production of salicylic acid	ABF, AREB, NAC, CBF CBF4, MYB, NAM, MYC, DREB1, REB2 (transcription factors)	(ROS) scavenging enzymes, PR proteins, 12-oxophytodienoate reductase	Alvarez et al., 2014; Kosová et al., 2014, 2015
Protein metabolism	Alterations in complete translational machinery along with protein biosynthesis	Elongation factor eEF-1α	E1 to E3 components ubiquitin ligase complex	Ghabooli et al., 2013; Kosová et al., 2015
Amino acid metabolism	Increased *S*-adenosylmethione, Phenylalanine, γ-aminobutyric acid (GABA), proline, tryptophan, tyrosine, phenylalanine, leucine, isoleucine, and valine	Methylation of monolignols	SAMS, PAL.	Bowne et al., 2012; Faghani et al., 2015; Shankar et al., 2016
Hormone metabolism	Upregulation of abiotic-stress-associated hormones such as JA, ABA, and SA	GA2OX1 (involved in gibberellin signaling), GID1L2 (gibberellin receptor involved in gibberellin signaling)	DELLA proteins, 9-*cis*-epoxycarotenoid-dioxygenase	Krugman et al., 2011; Kosová et al., 2015; Shankar et al., 2016
Energy metabolism	Rise and fall in the levels of various proteins related to respiration, ATP-biosynthesis, and respiration	RubisCO LSU, PSI Fe-S, PSII LHC protein, and SSU (photosynthesis related transcripts)	PGK, PRK, RubisCO activase, pyruvate kinase, alcohol dehydrogenase, and 2,3-bisphosphoglycerate-independent phosphoglycerate mutase.	Kosová et al., 2015; Vítámvás et al., 2015
Stress-responsive proteins	Increased deposition of hydrophilic proteins and osmolytes with chaperone functions	GABA and polyamines, dehydrin protein DHN5	HSP70, HSP90, HSP100, PDI, P5CS	Vítámvás et al., 2015; Bevan et al., 2017
Cellular transport	Variation in protein ingredients determining both membrane and cytoplasmic transport		Actin, Annexins	Zhang et al., 2014; Vítámvás et al., 2015
Metabolic activities monitoring the cell wall	Disruption in the metabolism of lignin and polyglucan, which is associated with reduced cell wall extensibility	Extensin, ABA, glycine-rich protein, and germin	XET, PAL, COMT, caffeoyl-CoA,	Krugman et al., 2011; Alvarez et al., 2014; Shankar et al., 2016
Recovery after stress	Transcripts of many drought-associated genes such as sugar transporters and protein kinases show downregulation	Cytochrome P450, COR410 SDi-6, HCF136, tubulin α-2, and OEE2	Polyubiquitin, peroxidases, P5CS, HSP60, and CCOMT	Ford et al., 2011; Hao et al., 2015
Mechanisms during grain-filling phase	Chlorophyll degradation in spike organs indicates a reduced oxidative owing to decreased rates of photosynthesis		Chlorophyllase, pheophorbide a oxygenase	Shankar et al., 2016; Vu et al., 2017

*Abbreviations for accessory proteins XET, 1,4-β-endo-transglycosylase; PAL, phenylalanine ammonia lyase; COMT, caffeic acid *O*-methyltransferase; P5CS, pyrroline-5-carboxylase synthase; PGK, phosphoglycerokinase; PRK, phosphoribulokinase; SAMS, *S*-adenosylmethionine synthetase; MAPK, mitogen activated protein kinase; CIPK, CBL-interacting protein kinase; HSP, heat shock proteins; CCOMT, Caffeoyl-CoA *O*-methyltransferase; GABA, gamma-aminobutyric acid; DHN5, Wheat dehydrin protein.*

Over the course of time, systems biology has appeared as a promising field that integrates massive amounts of data from genome-wide technologies and involves the use of computational models to help understand the topology and dynamical function of the molecular systems that constitute and sustain an organism (Noctor et al., 2014; Pandey et al., 2017). A large number of collaborating networks of responses have been constructed for model plants under abiotic stresses. The main objective of this review is to elucidate the molecular dynamics of wheat under drought and salinity stress, as well as to develop comprehensive stress-signaling models that can integrate stress systems biology with omics. Various cellular processes and antioxidant mechanisms operate inside the cell system to counter the alterations induced in cellular homeostasis by drought and salinity (Pandey et al., 2017; Zang et al., 2017). Therefore, before discussing how various mechanisms act under such circumstances, we need to understand the basic dynamics of working systems to address the homeostatic alterations in wheat systems biology.

## Antioxidant Systems: Defense, Signaling, and Stress Regulation

The production of intracellular ROS (reactive oxygen species) under optimal growth conditions are reactive chemical species. Under abiotic stress conditions, the CO_2_ uptake is limited, which causes stomatal closure and favors the photorespiratory production of superoxides, singlet oxygen, and H_2_O_2_ in the peroxisome due to over reduced photosynthetic electron transport chain (Noctor et al., 2014). Plasma membrane and the apoplast are the main sites for ROS generation in response to various exogenous environmental stimuli and endogenous signals. Hyper production of ROS under abiotic stresses cause extensive deregulation of cellular energetics and inhibition of physiological processes in plants, which further effects plant growth and yield. These overproduced ROS are highly reactive and toxic for the breakdown of proteins, lipids, and nucleic acids with a result in cell death and could also work as signals for the activation of stress response pathways (Gill and Tuteja, 2010; Baxter et al., 2014; You and Chan, 2015). To protect these cellular damages in plants against these overproduced ROS, an efficient enzymatic and non-enzymatic antioxidative system exists to modulate these ROS at low levels for signal transduction pathways. A dynamic equilibrium between ROS production and scavenging is usually disturbed when ROS production overwhelms the cellular scavenging capability (Pandey et al., 2017). This disequilibrium results in a sudden excess of ROS, commonly called oxidative stress (Zang et al., 2017). In these circumstances, antioxidative mechanism would be an instantaneous endogenic choice for the plants to counter ROS hyper production, under abiotic stresses which cause high ROS concentration and cellular damage inside the cell.

Plants possess antioxidant machinery for ROS scavenging and the protection of cells from oxidative damage. To sustain growth, production, metabolism, and development, as well as to overcome the potential damage by ROS to cellular parts, the balance between ROS generation and scavenging should be firmly regulated (Tang et al., 2014; Zang et al., 2017). This balance is maintained by both enzymatic and non-enzymatic antioxidants (Tang et al., 2014). Enzymatic antioxidants include glutathione reductase (GR), peroxidase (POX), glutathione peroxidase (GPX), ascorbate peroxidase (APX), catalase (CAT), superoxide dismutase (SOD), dehydroascorbate reductase (DHAR) and mono dehydroascorbate reductase (MDHAR) (Varga et al., 2012; Tang et al., 2014), whereas non-enzymatic antioxidants include phenolic compounds such as glutathione (GSH), carotenoids, tocopherol and ascorbate (Varga et al., 2012). Antioxidant enzymes work together to detoxify ROS and located at different sites within plant cells. Initially, the SOD antioxidant convert O_2_ into H_2_O_2_ and later on, CAT, APX, and GPX enzymes detoxify the H_2_O_2_ generated in the first step. Unlike CAT, APX needs non-enzymatic antioxidants such as GSH and ascorbic acid to reduce H_2_O_2_ with the help of MDHAR, DHAR, GR, GPX, and GST (Varga et al., 2012; Keunen et al., 2013). Conversely, PRX and organic hydroperoxides use the GSH, thioredoxin (TRX), or glutaredoxin (GRX) as nucleophiles through ascorbate-independent thiol-mediated pathways (Keunen et al., 2013). Non-enzymatic antioxidants are also crucial for ROS homeostasis in plants and include carotenoids, flavonoids, GSH, AsA, and tocopherols (Tang et al., 2014). Besides traditional enzymatic and non-enzymatic antioxidants, soluble sugars, including raffinose, fructose, glucose including various disaccharides and oligosaccharides, also have a role with respect to ROS detoxification (Keunen et al., 2013). The production rates of ROS are directly linked with soluble sugars, which regulate mitochondrial respiration or photosynthesis metabolic pathways to detoxify ROS (Pandey et al., 2017). On the other hand, they also feed NADPH-producing metabolic pathways to contribute to antioxidative progressions (Kong et al., 2013; Caverzan et al., 2016).

Avoiding ROS production under abiotic stress conditions might also be more important to maintain ROS homeostasis than the antioxidative system (You and Chan, 2015). In the electron transport chains of mitochondria, the excess generation of ROS can be prevented by alternative oxidases (AOX) (Niu and Liao, 2016). AOX pathway can also decrease the electron leaking possibility to O_2_ to generate super-oxide by diverting the electrons flowing through electron-transport chains. Additional mechanisms, such as the rearrangement of the photosynthetic apparatus, leaf movement, and curling might also signify an effort to avoid the over-reduction of ROS by harmonizing the amount of energy absorbed by the plant as well as CO_2_ availability (Mittler, 2002). In wheat, alterations in the activity of antioxidant enzymes POX, SOD, CAT, APX, and GR (Caverzan et al., 2016), and in the level of ROS (Kong et al., 2013) during abiotic stress have been reported to counter oxidative stress (Talaat and Shawky, 2014). Correspondingly, these findings reveal activation of the ROS scavenging processes in wheat. The alteration in the antioxidant activity of these enzymes in wheat is a defense mechanism to avoid oxidative damage under abiotic stress (Xu et al., 2013). High concentrations of ROS are deleterious to the plant, and therefore, the activation of both enzymatic and non-enzymatic entities triggers redox homeostasis to eliminate toxic levels of ROS (Varga et al., 2012). However, studies have revealed that different genotypes of wheat show differential responses to the same stress condition. The higher antioxidant ability of tolerant genotypes is related to their genetic architecture and protects them from severe oxidative damage. Furthermore, the complexity of ROS production and scavenging mechanisms in wheat is determined by the length and intensity of stress, as well as the developmental stage and tissue type.

Similarly, the stress type, intensity, and duration also regulate the production of H_2_O_2_, and its concentration differs across various cellular compartments (Talaat and Shawky, 2014). In biological systems, H_2_O_2_ is one of the most abundant ROS; it causes high toxicity due to its high reactivity (Xu et al., 2013). It is a signaling factor that triggers various responses in plant cells to counter abiotic stresses. Several factors such as production site, type of stress, and exposure time, as well as concentration, determine the biological effect of H_2_O_2_ (Petrov and Van Breusegem, 2012; Xu et al., 2013). At low concentrations, H_2_O_2_ serves as a signaling molecule, owing to its ability to diffuse across plasma membranes and its compartmentalization in cellular organelles, and thus elicits the stress response in crop plant (Petrov and Van Breusegem, 2012). Recent studies have revealed that, in wheat, early H_2_O_2_ treatment improves tolerance to abiotic stresses; however, these responses are not completely elucidated in adult plants at their final growth stages (Petrov and Van Breusegem, 2012; Talaat and Shawky, 2014). Studies conducted on the stress physiology of biological systems have demonstrated varying physiological responses at different developmental stages. Ge et al. (2013) have reported that H_2_O_2_ acts as both a signaling molecule and a deleterious agent in wheat seedlings under stress. Correspondingly, the concentration of H_2_O_2_ determines its beneficial or toxic role in plants. Following H_2_O_2_ signaling, various signaling entities such as miRNAs, transcription factors, and MAP-kinases participate in transduction networks (Petrov and Van Breusegem, 2012). Moreover, H_2_O_2_ production sites, concentrations, and crosstalk with other signaling pathways also play an important role in determining the subsequent response (Ge et al., 2013). Hence, the processes by which ROS scavenging counters different stresses need to be investigated further as several other biochemical, genetic, and molecular pathways could be involved in and contribute to this tolerance (You and Chan, 2015).

## Salinity Tolerance: Signaling, Gene Expression, and Regulation Prototype

Wheat plants utilizes phenotypic plasticity to mitigate the effects of salinity stress by upregulating of various stress responsive genes including ion transporters, transcriptional factors, signaling pathway modifiers, osmolytes production and antioxidative enzymes (Ge et al., 2013). Numerous pathway responses that altered due to the salinity mark the salt-responsive genes in tolerant plants which facilitate to understand the expression prototype of existing genes during the whole span of stress (Darko et al., 2017). Many genes are implicated in salinity tolerance; however, a comprehensive investigation is needed to resolve the complexity of the response to salinity stress at the genomic level (Abogadallah, 2010; Darko et al., 2017). Unification of systems biology and omics could specifically elucidate the genomic and metabolic responses of cells in a precise manner, providing better insights into various interconnecting signaling process that regulate cellular homeostatic machinery during stress.

High salinity level creates ionic imbalance and hypertonic effects, which inhibit crop yield at the molecular, biochemical, and physiological levels, either directly or indirectly (Abogadallah, 2010). Moreover, salinity stress is predicted to hamper photosynthesis, enhance photorespiration, deactivate enzymes, increase ROS damage, and ultimately lead to chloroplast damage. Hence, plants have developed various processes, including salt exclusion and compartmentalization (Zhang et al., 2016), to effect the successive biological and physiological changes that mitigate the harmful effects of salt stress (Darko et al., 2017). This phenotypic plasticity is governed by the upregulation and downregulation of different genes to decrease or protect from ROS damage, reformulate osmotic and ionic balance, and resume growth during high levels of salinity stress (Liu et al., 2014).

To elucidate the molecular dynamics of salt tolerance and increase the productivity of crops, substantial efforts has been made to develop genetic model systems. ROS scavenging is an efficient means mitigate oxidative damage and manipulate the expression of associated genes such as those encoding SOD, APX, and GRs, which provide salt tolerance (Abogadallah, 2010). Vacuolar compartmentalization maintains Na^+^/K^+^, and thereby also enhances salt tolerance.

**FIGURE 1:**
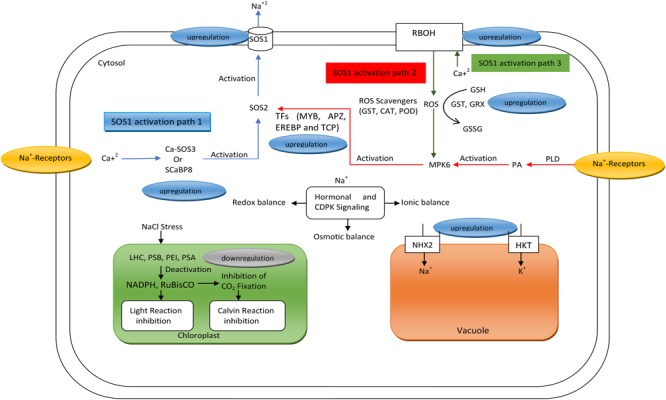
Model depicting the regulation of genes during different pathways to counter the effects of salinity in wheat leaf cells. (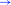) Indicting first pathway involved in the activation of SOS1, (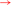) Indicating second pathway involved in the activation of SOS1, (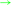) indicating third pathway involved in the activation of SOS1, (→) Indicating the occurrence of systematic processes in cytosol, chloroplast and vacuole under salt stress. RBOH, respiratory burst oxidase homologs; SOS, salt overly sensitive; SCaBP8, SOS3-like calcium binding protein8; GST, glutathione *S*-transferase; CAT, Catalase; POD, peroxidase; GRX, glutaredoxins; CDPK, calcium-dependent protein kinase; MPK6, mitogen-activated protein kinase6; PA, phosphatidic acid; PLD, Phospholipase D.

Zhang et al. (2016) reported three pathways governing the salinity counter mechanisms in cotton. However, we proposed a salinity counter model for wheat that demonstrates how various genetic determinants are regulated via different pathways, ultimately leading to cellular homeostasis (**Figure 1**). The cell membrane is equipped with proteins serving as Na^+^ receptors, which receive stress signals and elicit the production of signaling entities like Ca^2+^, ROS, and hormones (Liu et al., 2014). The elevated levels of these entities trigger three pathways that ultimately activate SOS1 to pump out Na^+^ from the cytosol. All the genes determining these pathways are upregulated. However, in this salinity counter mechanism, the photosynthesis process is inhibited, as RuBisCO and NADPH are deactivated (Zhao et al., 2014) owing to the down regulation of genes like LHC, PSB, PEI, and PSA (Liu et al., 2014).

However, an excess of Na^+^ hinders the uptake of K^+^ and cytosolic enzymes (Chao et al., 2013). The activity of Na^+^ and K^+^ transporters and H^+^ pumps and SOS2 and SOS3 protein kinase pathways coordinates with SOS1 to trigger the sequestration and secretion of toxic Na^+^ in the cell (**Figure 1**). Therefore, salt-tolerant genotypes resume growth at a slow rate when subjected to salt stress, owing to regulation by hormones and cell-division related genes. Increased deposition of ABA in response to salt stress is thought to upregulate cyclin-dependent protein kinase inhibitor (ICK1), which inhibits cell division (Wilkinson and Davies, 2010; Lee and Luan, 2012; Liu et al., 2015). Hence, these interconnected features constitute a breeding target for breeders to improve the potential range of adaptability of their germplasm to salt stress. The salinity tolerance of crop plants such as wheat is a multigenic trait, which is more complicated than in the model plant *Arabidopsis*, in addition to a high sensitivity to salinity (Shankar et al., 2016). Therefore, it is logical to conclude that wheat employs a more complicated system in response to salinity than Arabidopsis (**Table 1**).

Various genes have been reported to play a significant role in response to salt stress in wheat. For example, SRO (Similar to Rcd-One) mediates ROS deposition and scavenging by regulating the expression prototype of NADPH dehydrogenase and NADPH oxidase, together with GSH-peroxidase and ascorbate-GSH. Dynamic expression of these genes authenticates their inevitability and sufficiency in enhancing salt tolerance (Liu et al., 2014; Zhao et al., 2014). Nevertheless, the processes mediating the genome-wide gene expression in wheat to control the deleterious effects imposed by salinity are still not completely understood. Moreover, it has been reported, using a microarray approach, that out of 32,000 detected ESTs in wheat, 19% were either up- or down-regulated (Kawaura et al., 2006, 2008).

The adaptability of plants to unfavorable environments has been also explained through polyploidization (Dubcovsky and Dvorak, 2007). For instance, tetraploid Arabidopsis has a greater tolerance to salt stress, via the homeostasis of K^+^ and Na^+^, than diploid Arabidopsis (Chao et al., 2013). However, the molecular mechanisms determining adaptability to environmental stresses via this route are still poorly understood. It has recently been hypothesized that the expression of homologous genes is responsible for increased tolerance to salt stresses in polyploid plants. For example, in allopolyploid cotton (*Gossypium hirsutum*), one copy of the alcohol dehydrogenase A gene (AdhA) is upregulated under cold conditions, while the other responds to water stress (Liu and Adams, 2007). Moreover, transcriptomic studies have reported that allohexaploid wheat manifests intensive partitioned expression of homeologs in response to drought and heat stress (Liu et al., 2015).

## Drought Tolerance: Signaling, Gene Expression, and Regulation Prototype

Wheat, with its large genome, is a genetically complex entity, and is hypothesized as an ideal system to investigate the signaling processes involved in mediating stress response (Kang and Udvardi, 2012). Successful chromosome-based draft sequencing in hexaploid wheat has facilitated the mining of genes that regulate these complex processes during drought signaling, further accelerating the breeding programs. Current developments in omics and systems biology would further help researchers to better understand the mechanisms that operate at a cellular level to mitigate drought stress (Wang et al., 2014). To date, many researchers have comprehensively described some of the molecular and physiological phenomena that help to mitigate drought stress in plants (**Table 1**). However, in wheat, the activation of various regulatory mechanisms, owing to different mediatory agents, leads toward the homeostasis of plant cell system.

Abscisic acid (ABA)-mediated signaling during drought stress leads to rapid stomatal closure that inhibits the loss of water from leaves (Wilkinson and Davies, 2010; Lee and Luan, 2012). Moreover, under drought conditions, reduced water potential leads to increased accumulation of ABA, which regulates stress-related downstream responses (Aprile et al., 2013). Two major responses, osmotic adaptation and an increase in the concentration of osmolytes such as glycine betaine, glutamate, proline, and sugars (trehalose, sorbitol, and mannitol), appear at the cellular and molecular level to nullify drought effects by preventing membrane deterioration and enzyme inactivation (Slama et al., 2015). Furthermore, many drought-responsive genes and specific protective proteins are regulated for drought resistance (Ford et al., 2011). Signal transduction pathways regulate drought-stress-associated transcripts, proteins, reactive oxygen species (ROS) scavengers, and antioxidants (Faghani et al., 2015). ROS scavenging pathways protect the cell from oxidative damage under drought stress. Antioxidant enzymes, such as glutathione *S*-transferase (GST), APX, SOD, GR, GPX, and CAT, participate in ROS scavenging (Ford et al., 2011; Faghani et al., 2015). THz upregulation of these enzymes under drought stress indicates the presence of a potential system in plant cells to cope with drought stress.

**FIGURE 2:**
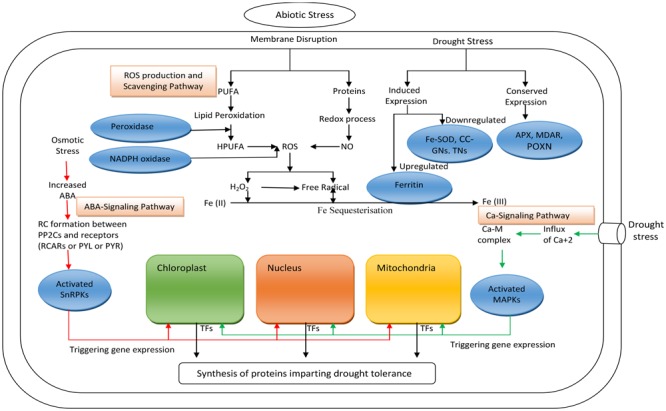
Systematic model showing the operational hierarchy of signaling pathways in wheat leaf cells that counter the effects of drought stress. (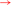) Indicating ABA mediated signaling pathway for protein synthesis, (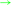) Indicating Ca^2+^ mediated signaling pathway for protein synthesis, (→) Indicating systematic changes involved in ROS production and scavenging pathway. PUFA, polyunsaturated fatty acids; HPUFA, hydroxy polyunsaturated fatty acids; ROS, reactive oxygen species; Fe-SOD, Fe-superoxide dismutase; CC-GNs, CC type glutaredoxins; TNs, thioredoxins; APX, ascorbate peroxidase; MDAR, monodehydroascorbate reductase; POXN, peroxiredoxin; SnRPKs, SNF1-related protein kinases; MAPK, mitogen-activated protein kinase; RC, regulatory component; PP2Os, Phosphatases type-2C; TFs, transcription factors.

In wheat, drought stress activates ROS generation and scavenging pathways and the Ca^2+^ and ABA signaling pathways, as depicted in (**Figure 2**). Under stress, genes manifest both induced and conserved expression. However, upregulation of ferritin plays an important role in ROS scavenging (Pizzio et al., 2013; Alvarez et al., 2014), as oxidation of Fe (II) to Fe (III) consumes H_2_O_2_ and oxygen (O_2_) (Kosová et al., 2015) during Fe sequestration.

Although, extensive research has been conducted on plants to better understand the induction of drought responsive mechanisms (Kosová et al., 2015), the process is still poorly understood, owing to the complicated nature of this quantitative trait (Ashoub et al., 2013). Until now, limited knowledge is available on the molecular mechanisms of drought tolerance in wheat genotypes. Drought-inducible proteins isolated from different wheat organs, such as roots, seedling, leaves, stem, and grains, have been revealed to be differentially expressed, and this differential expression is responsible for the drought resistance of tolerant genotypes (Vítámvás et al., 2015). Proteome regulation in wheat takes place in three phases (Kosová et al., 2014) that are indicated in along with their categories, potential consequences, and signaling proteins (**Table 2**).

**Table 2:** Phases of proteome regulations in wheat under drought stress with their categories, potential consequences, and signaling.

Proteome phase	Categories	Consequences	Signaling proteins	Reference
Alarm phase	Stress signaling and gene Expression	Alterations in physiochemical characteristics of plasma membrane. Phytohormones like ABA, JA, SA, and others show upregulation	G-proteins, PLC, PLD, MAPK, CDPK, PP2C, Aquaporins	Alvarez et al., 2014; Kosová et al., 2015; Montenegro et al., 2017
Acclimation phase	Protein metabolism	Regulatory changes in cell cycle and programmed cell death (PCD). Metabolic activities associated with protein degradation and biosynthesis show continuous alterations	eIF5A, TCTP, SAM, IDI2, IDS2, IDS3	Kosová et al., 2014, 2015
	Energy metabolism	Changes in various protein metabolisms have direct impacts on energy metabolism. A fall in the levels RuBisCO as well as Calvin cycle enzymes PRK, PGK, and transketolase.	OEE1, OEE2, CPN60-α, CPN60-β, TPI, 20-kDa, GAPDH, Enolase, β-conglycinin	Kosová et al., 2015; Mostek et al., 2015; Cheng et al., 2016
Resistance phase	Stress-protective proteins	Improper protein folding of HSPs due to the absence of hydration envelopes. Upregulation of Protein disulfide isomerase. Rise in ROS-scavenging enzymes increases the risks of protein damage	HSP110, HSP90, HSP70, HSP60, GDC, NADP-ME3,NADP-ME4,TSI-1 protein	Fercha et al., 2014; Kosová et al., 2015; Mostek et al., 2015
	Structural proteins	Cellular transport and cytoskeleton get impaired profoundly. An increase in aquaporin proteins and its differential phosphorylation. Rate of cell division and plant growth decrease significantly. Increased cell wall lignification	VDAC, SAM, CCOMT, COMT.	Witzel et al., 2014; Kosová et al., 2015; Cheng et al., 2016

*PL, phospholipases; MAPK, mitogen activated protein kinase; CDPK, calcium-dependent protein kinase; PP, protein phosphatase; 5A/eIF5A, eukaryotic translation initiation factor; TCTP, translationally controlled tumor protein homolog; SAM, *S*-adenosylmethionine; OEE, oxygen evolving enhancer (protein); CPN, Chaperonin; TPI, triose phosphate isomerase; GAPDH, glyceraldehyde-3-phosphate dehydrogenase; GDC, glutamate decarboxylase; ME, malic enzyme; VDAC, voltage-dependent anion channel.*

Pioneering transcriptome studies have documented that the drought-sensitive and tolerant genotypes of wheat are equipped with different molecular mechanisms to mitigate drought stress (Mohammadi et al., 2007; Aprile et al., 2013). A number of drought-related genes showing constitutive expression in tolerant wheat genotypes are also known to be triggered in drought-sensitive genotypes, and such expression is a limiting attribute in the understanding of response mechanisms induced by drought (Aprile et al., 2013). Moreover, hormonal and enzyme-based regulation pathways show variations in different wheat genotypes (Ergen and Budak, 2009). When tolerant genotypes are affected by drought stress, prompt activation of signal transduction pathways triggers downstream elements. Differential response of specific transcription factors in different wheat genotypes indicates the presence of different signaling pathways mediated by hormones. The induction of transcription factors that bind to ethylene-responsive elements has been reported in a sensitive wheat genotype, whereas the induction of bZIP and HDZIP genes transcription factors related t to ABA regulation has also been reported in tolerant wheat genotypes under drought stress (Ergen and Budak, 2009). To date, these studies have provided a significant evidences about signaling dynamics in response to drought stress; however, the transcriptional responses are not sufficient to estimate post-transcriptional and post-translational modifications (Pradet-Balade et al., 2001). Moreover, little is known about the functional outputs of these detected genes, and hence, it is difficult to establish the relationship between transcriptome and proteome in drought-sensitive and tolerant wheat genotypes under stress.

Currently proteomics is becoming the most dynamic and direct accessory to unravel the function of expressed proteins under drought stress (Ford et al., 2011; Ghabooli et al., 2013). It can be complemented by transcriptome studies to generate a global expression profile of proteins encoded by the genome (Bowne et al., 2012; Vu et al., 2017). Comparative proteome profiling of tolerant and sensitive genotypes could also help to explain the complexity of induced molecular processes in wheat during drought stress (Krugman et al., 2011). To date, only a few studies have been conducted to examine the proteomic alterations under stress in wheat genotypes (Bevan et al., 2017; Vu et al., 2017).

## Conclusion

Exploitation of the mysterious genomic attributes that impart tolerance to abiotic stresses in wheat is a potential challenge for scientists. Although substantial efforts have been made in this direction, several research gaps need to be fulfilled. Therefore, integration of stress systems biology with recent omics approaches would be helpful in unraveling the potential mechanisms involved in countering abiotic stresses. This would provide a robust and focused dimension to crop improvement programs.

## Author Contributions

ZS, HR, MN, MA and TA came with idea and wrote the manuscript. ID, RA, and IR are reviewed and SY and GC critically analyzed the manuscript.

## Conflict of Interest Statement

The authors declare that the research was conducted in the absence of any commercial or financial relationships that could be construed as a potential conflict of interest.
